# Supplementary material for: A 2018 Reference Guide to the Banff Classification of Renal Allograft Pathology
Source: Transplantation. 2018 Oct 26;102(11):1795–814. doi: 10.1097/TP.0000000000002366 (PMC7597974; doi:10.1097/TP.0000000000002366)
Supplement: SUPPLEMENTARY MATERIAL [file tp-102-1795-s001.pdf]

SDC, Glossary of terms

Acute Tubular Injury

There is no current definition of acute tubular injury endorsed by the Banff classification. In the absence of other apparent cause (Banff 2015), acute tubular injury is included as one of the criterion for histological evidence of acute tissue injury in the diagnosis of acute/active ABMR.(12) Moreover, it is included as a Diagnosis in Banff Diagnostic Category 6.

Antibody-mediated rejection (ABMR)

Antibody-mediated rejection (ABMR) refers to a rejection process believed to be primarily driven by antibodies against graft epitopes. Diagnostic criteria are listed under Banff Diagnostic Category 2 of the 2017 Banff update.(12) Diagnostic subcategories within include the following; C4d Staining Without Evidence of Rejection, Active ABMR, Chronic Active ABMR, Chronic ABMR. ABMR can coexist with additional diagnoses from Banff classification categories 3-6.

Adequacy of Specimen

Since Banff 1997(5) a biopsy has been considered adequate if it contains at least 10 or more glomeruli and at least 2 arteries; the threshold for a “minimal sample” is 7 glomeruli and 1 artery. It is also recommended that at least 2 separate cores containing cortex be obtained or that there be 2 separate areas of cortex in the same core.(11)

Arteriole

Derived from the definition of arteries below, the definition of arterioles is consequently arterial vessels having less than 2 smooth muscle layers.(11) Changes in arterioles are currently not recognised as contributory to Banff Lesion Score v or cv.(12) Arteriolar inflammation is noted with an asterisk behind Banff Lesion Score ah, eg, ah2*.(5)

Arteritis, Intimal

Arteritis is synonymous with endarteritis or endothelialitis. According to Banff 2015, intimal arteritis as per Banff Lesion Score v1, v2 is defined as mononuclear cell infiltration beneath the arterial endothelium. Severe arteritis v3 is defined by inflammation in the media and/or fibrinoid necrosis of the vessel wall; the total number of arteries in the biopsy and the number of arteries affected should be noted.(11) Marginated leukocytes alone are insufficient to diagnose arteritis.

Artery

Since Banff 2013(10) an artery has been defined by “having a continuous media with two or more smooth muscle layers”.(11)

Borderline Changes

This refers to Banff Diagnostic Category 3 “Suspicious (Borderline) for Acute TCMR”.(12) This can be used in conjunction with other Banff categories with the exception of category 1 (Normal biopsy or nonspecific changes) or 4 (Acute TCMR Grade IA, IIA, IIB or III, Chronic active TCMR Grade IA, IB or II).

C4d

Complement component 4 fragment d (C4d) is a degradation product of complement component C4, which can bind covalently to the endothelial cell surface.(30) Positive immunostaining indicates local complement activation and is scored with Banff lesion score C4d.(11) The clinical significance of these findings is different in grafts exposed to anti-blood group antibodies (ABO-incompatible allografts), in which isoagglutinin-mediated complement activation does not appear to be injurious to the graft and may represent accommodation. However, when caused by antibodies against donor-specific HLA or other antigens, it may indicate ABMR activity. Based on the high specificity of C4d positivity, it is considered equivalent to the serological proof of donor-specific antibody (DSA) for a diagnosis of ABMR, although it is still recommended to test for DSA.

Banff Diagnostic Categories

This refers to the 6 consensus-based and empirically validated Banff Diagnostic Categories (1 to 6) for renal allograft biopsies.(12) Diagnoses from different Banff Diagnostic Categories can be used if required in any combination other than category 1, 3 and 4 which are mutually exclusive. Moreover, any diagnosis from categories 2-6 excludes Banff Diagnostic Category 1 (Normal biopsy or nonspecific changes)[9]. Multiple diagnoses from a single Banff Diagnostic Category can only be made from Category 6.

Banff Lesion Scores

Banff Lesion Scores are descriptive, consensus-based, standardised terms to grade important active and chronic histopathological findings in the different morphological compartments of the renal transplant. Although they are an integral part of the minimal dataset, they are by themselves not necessarily sufficient to determine Banff Diagnostic Categories.

Chronic Allograft Arteriopathy

Chronic Allograft Arteriopathy is a feature of Chronic Active TCMR, Chronic Active ABMR, Chronic ABMR and Chronic Active ABMR. It is defined as arterial intimal fibrosis with mononuclear cell infiltration in fibrosis and/or formation of neointima. The Banff lesion score cv is used to grade it, based on the extent of luminal occlusion in the most severely affected artery. Lack of prior biopsy-proven TCMR favours a diagnosis of Chronic Active ABMR or Chronic ABMR.(12)

Cortex

The outer part of the renal parenchyma, beneath the capsule and peripheral from the medulla, including the columns of Bertin. It is characterised by the presence of glomeruli and arteries.(31)

Donor-specific Antibody (DSA)

Circulating antibodies directed against donor human leukocyte antigen (HLA) class I and/or class II and/or non-HLA antigens. For the definition and technical details see the recent and forthcoming consensus recommendations by The Transplantation Society.(13) DSA are relevant for Banff Diagnostic Category 2 (ABMR) diagnoses. For Active and Chronic Active ABMR only DSA present at the approximate time of biopsy and against the current

transplant are considered positive for the diagnosis; for Chronic ABMR DSA against the current transplant present at or prior to the biopsy are considered.(12)

Fibrointimal Thickening, Arterial

See → Transplant Arteriopathy.

Gene Transcripts/Classifiers in the Biopsy Tissue strongly associated with ABMR

Increased Expression these, if thoroughly validated, are the only molecular markers considered in the Banff classification. Since Banff 2013(10) they have been included in Banff Diagnostic Category 2 for the diagnosis of Active ABMR and Chronic Active ABMR.(11) Along with C4d positivity, they are considered equivalent to positive test for DSA, although serological testing for DSA is still encouraged.(12)

Glomerulonephritis

Recurrent or de novo glomerulonephritis can occur in renal transplant biopsies. The diagnosis is made by a combination of conventional histology, immunohistochemistry or immunofluorescence, electron microscopy and correlation with clinical data. It should be diagnosed under Banff Diagnostic Category 6, and can be used in combination with categories 2-5 and any other diagnosis from Category 6. The presence of glomerulonephritis precludes the use of Banff Lesion Score g as histologic evidence of acute tissue injury for the diagnosis of Active ABMR and of Banff Lesion Score cg as evidence of chronic tissue injury in the diagnosis of Chronic Active or Chronic ABMR.(12)

Hemorrhage, Tubulointerstitial

Extravasation of red blood cells into the tubulointerstitium. There is no special Banff Lesion Score for this lesion. Instead the lesion is noted as an asterisk "*" attached to Banff Lesion Score v (eg, v0* or v3*). It shares this coding with Infarct.

Infarct

Zone of tissue which is nonviable due to insufficient blood supply. It qualifies as "necrosis" listed in the supplement of Banff 2015 and since Banff 1997 (Table 3) (5) has been coded with an asterisk (*) behind any of the Banff Lesion Score v (eg, v1*) accordingly.(11) Infarcted areas must not be scored for Banff Lesion Score C4d.(8)

Infection

In the presence of Infection (Table 5, Banff 2017) Banff Lesion Score g>0 greater is required when using moderate microvascular inflammation (sum of Banff g+ptc≥2) as a criterion from Criteria Group 3 (Antibody interaction with tissue) for a diagnosis of Active ABMR or Chronic Active ABMR.(12) Ie, Banff Lesion Score ptc2 with Banff Lesion Score g0 is insufficient for this criterion when disease caused by infection of the transplant is present.

Interstitial fibrosis

Banff Lesion Score ci for interstitial fibrosis was initially meant to describe the area fraction of fibrous tissue in the cortex only regardless of whether it represented fibrous tissue as the normal constituent of the renal cortex (considered to be 5%) or fibrous tissue within a cortical scar. Hence, a precise definition for individual patches of interstitial fibrosis in a scar

has never been given. This purely morphometric approach runs counterintuitive to routine histopathological assessment for atrophic tubules separated by interstitial fibrosis in a tubulointerstitial scar. Guides for the assessment of Banff Lesion Score ci can be found in the main body of this manuscript.

Microvascular Inflammation, Moderate

Microvascular inflammation is scored by calculating the sum of the 2 Banff Lesion Scores g and ptc. Microvascular inflammation is considered moderate with [Banff Lesion Scores g + ptc] ≥2. In the presence of Acute TCMR, Borderline Changes or Infection such as, Pyelonephritis, Banff Lesion Score g must be equal or greater than 1 in this calculation to fulfil the criteria for moderate microvascular inflammation.

Peritubular Capillary

Only capillaries within the renal cortex are peritubular capillaries (PTCs) which can be used for scoring peritubular capillaritis as in Banff Lesion Score ptc. Capillaries next to lymphoid follicles must be disregarded to avoid confusion with lymphatic vessels. Banff C4d can be scored not only in PTCs but also in medullary vasa recta.

Peritubular Capillary Basement Membrane Multilayering

Severe Peritubular Capillary Basement Membrane Multilayering (PTCML) is defined as "seven or more layers of basement membrane in one cortical peritubular capillary and five or more in two additional capillaries, avoiding portions cut tangentially".(12) Only Severe Peritubular Capillary Basement Membrane Multilayering is considered sufficient as one possible criterion for chronicity of ABMR. This relative high threshold has been established because mild PTCML can be seen in other diseases like hypertension. In cases where EM is carried out in transplant biopsies, cortical peritubular capillary multilayering should be assessed and reported. The number of layers of basement membrane should be counted in the most affected PTC as well as in at least 2 additional PTCs. Tangentially cut PTC should be avoided. See also recommendations for performing EM cited from the Banff 2015 report under Banff Lesion Score cg for more detail.(11)

Pyelonephritis

Inflammation of the transplant parenchyma caused by bacterial or fungal infection, usually ascending from the bladder. This falls within the remit of Banff Diagnostic Category 6. As an →Infection, the presence of Pyelonephritis has important bearings on the diagnosis of Active ABMR and Chronic Active ABMR, as then Banff Lesion Score g must be equal or greater than 1 to fulfil the criteria for →Microvascular inflammation, Moderate ([Banff Lesion Score g + ptc] ≥2).(12)

Polyomavirus Nephropathy

Replicative infection of the kidney by polyoma virus as indicated by viral inclusions seen on histology, immunohistochemistry and/or electron microscopy. A Banff Working Group for Polyomavirus Nephropathy has developed and validated a respective scoring schema, which was presented at the 2017 and has been published recently. The percentage of tubules with replicative BK virus infection and Banff Lesion Score ci informs the classification from 1 to

3.(32) Polyomavirus nephropathy is included in Banff Diagnostic Category 6 as BK-Virus Nephropathy.

Scarred Cortical Parenchyma

Characterised by cortical tubular atrophy, usually but not always accompanied by interstitial fibrosis –see →Tubular Atrophy. Banff Lesion Score i must not be scored in these areas, instead Banff Lesion Score i-IFTA is assessed in the areas.

Subcapsular Cortex

This term refers to areas immediately beneath the capsule, which may show areas of nonspecific scarring or inflammation thought to be related to surgical 'healing in'.(1) The extent of the cortex that qualifies as "subcapsular" has not been more clearly defined in the Banff Classification. Banff Lesion Scores ptc(7) and i(5) must not be scored in these areas.

T cell-mediated rejection (TCMR)

The spectrum of TCMR is defined as Banff Diagnostic Category 4 which contains Acute TCMR Grade IA, IB, IIA, IIB, III as well as Chronic Active TCMR Grade IA, IB and II. Suspicious (Borderline) for Acute TCMR is the only diagnosis in Banff Diagnostic Category 3. Banff Diagnostic Categories 3 and 4 are mutually exclusive but can be rendered together with other diagnoses from Categories 2, 5 and 6.

Thrombotic Microangiopathy, Thrombotic Microangiopathy In The Absence Of Any Other Cause

Thrombotic microangiopathy (TMA) can be caused by recurrent disease (usually atypical hemolytic-uremic syndrome), ABMR, Calcineurin Inhibitor Toxicity (included in Banff Diagnostic Category 6) and other causes.(33) If there is evidence of chronic TMA of documented cause other than ABMR, Banff Lesion Score cg must not be used as a criterion for histological evidence of chronic tissue injury in the diagnosis of either Chronic Active ABMR or Chronic ABMR.(12)

Transplant Arteriopathy

Transplant Arteriopathy is defined as arterial fibrointimal thickening, also referred to as vascular fibrous intimal thickening, and is graded based on the extent of luminal occlusion in the most severely affected artery. Transplant arteriopathy is scored with the Banff Lesion Score cv. Features that suggest Chronic Active TCMR Grade II, Chronic Active ABMR or Chronic ABMR versus other causes such as conventional arteriosclerosis or hypertension are disruption of the elastica, inflammatory cells in the fibrotic intima, proliferation of myofibroblasts and/or foam cells in the expanded intima, formation of a neointima.(11) However, it should be noted that bland but progressive intimal fibrosis may be associated with the presence of DSA.(34)

Transplant Glomerulopathy

Transplant glomerulopathy is defined as a Banff Lesion Score cg greater than 0, after the exclusion of thrombotic microangiopathy of cause other than ABMR, eg, recurrent or de novo Glomerulonephritis. It is not synonymous to ABMR, but is frequently associated with it.

Tubular Atrophy

According to Banff 1997, tubulitis shall not be counted in moderately-to-severely Moderately atrophic tubules are defined as tubules having a diameter <50% of that of unaffected or minimally affected tubules on the biopsy, with wrinkling and/or thickening of the tubular basement membrane. (5)

Severely atrophic tubules are defined as tubules of diameter <25% of that of unaffected or minimally affected tubules on the biopsy, often with an undifferentiated-appearing, cuboidal or flattened epithelium (or in some cases even loss of epithelium with denudation of the tubular basement membrane), and pronounced wrinkling and/or thickening of the tubular basement membrane. This definition of severely atrophic tubules also includes very small, endocrine-like tubules with very narrow lumens, although the basement membranes of the latter may not be thickened.(12)

According to Banff 2017, severely atrophic tubules should be disregarded for Banff Lesion Score t.(12)

References

1. Solez K, Axelsen RA, Benediktsson H, et al. International standardization of criteria for the histologic diagnosis of renal allograft rejection: the Banff working classification of kidney transplant pathology. *Kidney Int*. 1993;44:411-422
2. Mengel M, Sis B, Halloran PF. SWOT analysis of Banff: strengths, weaknesses, opportunities and threats of the international Banff consensus process and classification system for renal allograft pathology. *Am J Transplant*. 2007;7:2221-2226
3. Becker JU, Chang A, Nickeleit V, Randhawa P, Roufosse C. Banff borderline changes suspicious for acute T-cell mediated rejection: where do we stand? *Am J Transplant*. 2016;16:2654-2660.
4. Solez K, Benediktsson H, Cavallo T, et al. Report of the Third Banff Conference on Allograft Pathology (July 20-24, 1995) on classification and lesion scoring in renal allograft pathology. *Transplant Proc*. 1996;28:441-444.
5. Racusen LC, Solez K, Colvin RB, et al. The Banff 97 working classification of renal allograft pathology. *Kidney Int.* 1999;55:713-723.
6. Racusen LC, Halloran PF, Solez K. Banff 2003 meeting report: new diagnostic insights and standards. *Am J Transplant*. 2004;4:1562-1566.
7. Solez K, Colvin RB, Racusen LC, et al. Banff '05 Meeting Report: differential diagnosis of chronic allograft injury and elimination of chronic allograft nephropathy ('CAN'). *Am J Transplant*. 2007;7:518-526.
8. Solez K, Colvin RB, Racusen LC, et al. Banff 07 classification of renal allograft pathology: updates and future directions. *Am J Transplant*. 2008;8:753-760.
9. Mengel M, Sis B, Haas M, et al. Banff 2011 Meeting Report: New Concepts in Antibody-Mediated Rejection. *Am J Transplant.* 2012;12:563-570.
10. Haas M, Sis B, Racusen LC, et al. Banff 2013 meeting report: inclusion of c4d-negative antibody-mediated rejection and antibody-associated arterial lesions. *Am J Transplant.* 2014;14:272-283.
11. Loupy A, Haas M, Solez K, et al. The Banff 2015 Kidney meeting report: Current challenges in rejection classification and prospects for adopting molecular pathology. *Am J Transplant*. 2017;17(1):28-41.

12. Haas M, Loupy A, Lefaucheur C, et al. The Banff 2017 Kidney Meeting Report: Revised Diagnostic Criteria for Chronic Active T Cell-Mediated Rejection, Antibody-Mediated Rejection, and Prospects for Integrative Endpoints for Next-Generation Clinical Trials. *Am J Transplant*. 2018;18:293-307.
13. Tait BD, Susal C, Gebel HM, et al. Consensus guidelines on the testing and clinical management issues associated with HLA and non-HLA antibodies in transplantation. *Transplantation*. 2013;95:19-47.
14. Liapis H, Gaut JP, Klein C, et al. Banff Histopathological Consensus Criteria for Preimplantation Kidney Biopsies. *Am J Transplant*. 2017;17:140-150.
15. Ishii Y, Sawada T, Kubota K, Fuchinoue S, Teraoka S, Shimizu A. Loss of peritubular capillaries in the development of chronic allograft nephropathy. *Transplant Proc*. 2005;37:981-983.
16. Farris AB, Chan S, Climenhaga J, et al. Banff fibrosis study: multicenter visual assessment and computerized analysis of interstitial fibrosis in kidney biopsies. *Am J Transplant.* 2014;14:897-907.
17. Baid-Agrawal S, Farris AB 3rd, Pascual M, et al. Overlapping pathways to transplant glomerulopathy: chronic humoral rejection, hepatitis C infection, and thrombotic microangiopathy. *Kidney Int*. 2011;80:879-885.
18. Olson JL. Renal Disease Caused by Hypertension. In: Jennette JC, Olsen SL, Silva FG, et al., eds. Heptinstall's Pathology of the Kidney. 7th Edition ed. Wolters Kluwer: Philadelphia, PA, USA; 2015, 849-896.
19. Mueller TF, Einecke G, Reeve J, et al. Microarray analysis of rejection in human kidney transplants using pathogenesis-based transcript sets. *Am J Transplant*. 2007;7:2712-2722.
20. Mannon RB, Matas AJ, Grande J, et al. Inflammation in areas of tubular atrophy in kidney allograft biopsies: a potent predictor of allograft failure. *Am J Transplant*. 2010;10:2066-2073.
21. Lefaucheur C, Gosset C, Rabant M, et al. T cell-mediated rejection is a major determinant of inflammation in scarred areas in kidney allografts. *Am J Transplant*. 2018;18:377-390.
22. Nankivell BJ, Shingde M, Keung KL, et al. The causes, significance and consequences of inflammatory fibrosis in kidney transplantation: The Banff i-IFTA lesion. *Am J Transplant*. 2018;18:364-376.
23. Solez K, Fung KC, Saliba KA, et al. The bridge between transplantation and regenerative medicine: Beginning a new Banff classification of tissue engineering pathology. *Am J Transplant*. 2018;18:321-327.
24. Solez K. Kim Solez, Edmonton, Alberta, Canada Banff: A Unique Start Setting Standards for Consensus Conferences. *Transplantation*. 2017;101:2264-2266.
25. Al-Awqati Q, Oliver JA. Stem cells in the kidney. Kidney Int 2002;61(2):387-395
26. Stubbington MJT, Rozenblatt-Rosen O, Regev A, Teichmann SA. Single-cell transcriptomics to explore the immune system in health and disease. *Science*. 2017;358:58-63.
27. Rozenblatt-Rosen O, Stubbington MJT, Regev A, Teichmann SA. The Human Cell Atlas: from vision to reality. *Nature*. 2017;550:451-453.
28. Regev A, Teichmann SA, Lander ES, et al. The Human Cell Atlas. *Elife*. 2017;6. doi:10.7554/eLife.27041.
29. Farris AB, Adams CD, Brousaides N, et al. Morphometric and visual evaluation of fibrosis in renal biopsies. *J Am Soc Nephrol*. 2011;22:176-186.

30. Regele H, Bohmig GA, Habicht A, et al. Capillary deposition of complement split product C4d in renal allografts is associated with basement membrane injury in peritubular and glomerular capillaries: a contribution of humoral immunity to chronic allograft rejection. *J Am Soc Nephrol*. 2002;13:2371-2380.
31. Bonsib SM. Renal Anatomy and Histology. In: Jennette CJ, Silva FG, Olson JL, et al, eds. Heptinstall's Pathology of the Kidney. Lippincot Williams & Wilkins: Philadelphia, PA, USA; 2015, 1-66.
32. Nickeleit V, Singh HK, Randhawa P, et al. The Banff Working Group Classification of Definitive Polyomavirus Nephropathy: Morphologic Definitions and Clinical Correlations. *J Am Soc Nephrol*. 2018;29:680-693.
33. Noris M, Remuzzi G. Thrombotic microangiopathy after kidney transplantation. *Am J Transplant*. 2010;10:1517-1523.
34. Hill GS, Nochy D, Bruneval P, et al. Donor-specific antibodies accelerate arteriosclerosis after kidney transplantation. *J Am Soc Nephrol*. 2011;22:975-983.
