# Supplementary material for: A 2018 Reference Guide to the Banff Classification of Renal Allograft Pathology
Source: Transplantation. 2018 Oct 26;102(11):1795–814. doi: 10.1097/TP.0000000000002366 (PMC7597974; doi:10.1097/TP.0000000000002366)
Supplement: SUPPLEMENTARY MATERIAL [file tp-102-1795-s002.docx]

A 2018 Reference Guide to the Banff Classification of Renal Allograft Pathology

Candice Roufosse^1^, MD, PhD

Naomi Simmonds^2^, MD

Marian Clahsen-van Groningen^3^, MD, PhD

Mark Haas^4^, MD, PhD

Kammi J. Henriksen^5^, MD

Catherine Horsfield^2^, MD

Alexandre Loupy^6^, MD

Michael Mengel^7^, MD

Agnieszka Perkowska-Ptasińska^8^, MD

Marion Rabant^9^, MD, PhD

Lorraine C. Racusen^10^, MD

Kim Solez^7^, MD

Jan U. Becker^11^, MD

# Affiliation

^1^ Dept. of Medicine, Imperial College, London, UK; North West London Pathology, London, United Kingdom

^2^ Department of Histopathology, Guy's and St. Thomas' National Health Service Foundation Trust, London, United Kingdom

^3^ Department of Pathology, Erasmus MC, Rotterdam, The Netherlands

^4^ Department of Pathology, Cedars-Sinai Medical Center, Los Angeles, CA, USA

^5^ Department of Pathology, University of Chicago, Chicago, IL, USA

^6^ Paris Translational Research Center For Organ Transplantation, U970 INSERM, Paris, France

^7^ Department of Laboratory Medicine and Pathology, University of Alberta, Edmonton, Canada

^8^ Department of Transplantology, Nephrology and Internal Diseases, Medical University of Warsaw, 02-006 Warsaw, Poland

^9^ Paris Translational Research Center for Organ Transplantation INSERM U970 & Necker Hospital University Paris Descartes, Paris, France

^10^ The Johns Hopkins University, Baltimore, MD, USA

^11^ Institute of Pathology, University Hospital of Cologne, Cologne, Germany

# Corresponding author

Jan U. Becker, MD

Institute of Pathology

University Hospital of Cologne

Kerpener Str. 62

50937

Germany

Phone: +4922147898803

Fax: +492214786360

E-mail: [janbecker@gmx.com](mailto:janbecker@gmx.com)

# Authors’ contribution

All authors contributed the discussion of the content, the collation of images and illustrations and to writing of the manuscript.

# Disclosure

The authors declare no conflicts of interest.

# Funding

Dr. Roufosse’s and Dr. Simmond’s contribution to this research is supported by the National Institute for Health Research (NIHR) Biomedical Research Centre based at Imperial College Healthcare NHS Trust and Imperial College London. The views expressed are those of the authors and not necessarily those of the NHS, the NIHR or the Department of Health.

Alexandre Loupy is supported by a research grant ATIP AVENIR from the National French Institute of Research.

Jan U. Becker is supported by the European Rare Kidney Disease Network (ERKNet); the content of this manuscript is the sole responsibility of the authors.

# Abbreviations

ABMR, antibody-mediated rejection

DSA, donor-specific antibody

EM, electron microscopy

HCAP, Human Cell Atlas Project

H&E, hematoxylin and eosin

IF, immunofluorescence

IFTA, interstitial fibrosis and tubular atrophy

IHC, immunohistochemistry

LM, light microscopy

MVI, microvascular inflammation

PAS, periodic acid-Schiff

PTC, peritubular capillary

PTCML, peritubular capillary basement membrane multilayering

TCMR, T cell-mediated rejection

TEP, tissue engineering pathology

TG, transplant glomerulopathy

TMA, Thrombotic microangiopathy

# Abstract

The Banff Classification of Allograft Pathology is an international consensus classification for the reporting of biopsies from solid organ transplants. Since its initial conception in 1991 for renal transplants, it has undergone review every 2 years, with attendant updated publications. The rapid expansion of knowledge in the field has led to numerous revisions of the classification. The resultant dispersal of relevant content makes it difficult for novices and experienced pathologists to faithfully apply the classification in routine diagnostic work and in clinical trials.

This review shall provide a complete and simple illustrated reference guide of the Banff Classification of Kidney Allograft Pathology based on all publications including the 2017 update. It is intended as a concise desktop reference for pathologists and clinicians, providing definitions, Banff Lesion Scores and Banff Diagnostic Categories. An online website reference guide hosted by the Banff Foundation for Allograft Pathology (www.banfffoundation.org) is being developed, which will be updated with future refinement of the Banff Classification from 2019 onwards.

# Introduction

Since its first consensus meeting in 1991,(*1*) the Banff Classification of Allograft Pathology has provided a framework for the reporting of renal allograft biopsies. It was the first classification system of its kind and answered the need for an international consensus on renal transplant biopsy reporting, providing guidance for clinical diagnosis and enabling meaningful comparison between research studies and clinical trials investigating the diagnosis, treatment and outcome in kidney transplantation. The Banff Classification has since been further strengthened by evidence-informed biannual updates elaborated during open international expert meetings.(*2*) As a result, the Banff Classification of Allograft Pathology has become the predominant classification system used worldwide.(*3*)

A total of 14 meetings reported in 10 manuscripts reflects the developments of the Banff Classification from the first consensus meeting in 1991 to the recently published consensus following the 2017 meeting in Barcelona, Spain.(*1, 4-12*) Each of these iterations provides a short summary of the meeting and contributes to the Classification in a cumulative fashion. The dispersal of both relevant and outdated content over 10 manuscripts could make access to the Banff Classification difficult for beginners and experts and has created ambiguities in the past.(*3*) Yet, accessibility and clarity are of utmost importance not only for clinical practice and research but also for the Banff Classification itself to evolve through accountability, critique and change. In order to improve on these aspects, the Rules and Dissemination Banff Working Group was initiated at the last Banff meeting held in Barcelona, Spain in March 2017. With a scope beyond the helpful syllabus provided by the Banff group in the online supplement of the 2015 update(*11*) and incorporating the latest changes introduced in the 2017 update,(*12*) the aim of this Working Group is to collate all current content of the Banff Classification and improve its accessibility. A systematic inventory of the content is given in Figure 1. This practical guide is based on all content up to the 2017 update as the first output of our Working Group. It is divided in the following sections: a brief guide about the histopathological and serological work-up; a list of Banff Lesion Scores (previously known as components, eg, Banff t for tubulitis) with their current definitions, practical tips for their application and illustrative figures (see definitions below and thresholds in Table 2); and a list of Banff Diagnostic Categories in Table 1. Moreover, we provide a list of Additional Diagnostic Parameters, which need to be considered in addition to Banff Lesion Scores in order to reach a Banff Diagnostic Category. (Table 3). Examples for these include “Severe Peritubular Capillary Basement Membrane Multilayering” which is among the criteria for antibody-mediated rejection (ABMR) chronicity.(*12*) A Glossary of Terms is provided as Supplemental Digital Content (see Glossary of Terms, SDC, http://links.lww.com/TP/B604), explaining important concepts and terminology underlying the Banff Classification. Lastly, we provide a critical appraisal of areas of the Banff Classification that require clarification and provide an outlook for future developments. All terms from the Banff Classification will be given in capitals for clarity, all abbreviations for Banff Lesion Scores will be given in italic typeface.

We hope this Banff 101 will serve as a handy reference for the clinicians and the pathologists, until the entire updated content appears online with the 2019 update of the Banff Classification of Renal Allograft Pathology, replacing this guide.

# Diagnostic Work-Up of Biopsies

A kidney transplant biopsy should fulfil the criteria for specimen adequacy (See Glossary of Terms, SDC, http://links.lww.com/TP/B604) detailed in the Banff 1997 update.(*5*) C4d staining is considered indispensable, either as immunofluorescence (IF) on fresh frozen or immunohistochemistry (IHC) on paraffin-embedded tissue. The paraffin block should be cut in several numbered level sections examined with hematoxylin-eosin, periodic acid-Schiff, trichrome-elastic and Jones or methenamine silver stains. IHC staining for simian virus-40, cross-reacting with BK virus is highly recommended when indicated. Where available, minute portions of cortex should be embedded for transmission electron microscopy (EM).

Depending on clinical and histopathological findings a complete nephropathological work-up including staining for immunoglobulin heavy- and light-chains and complement split products might be necessary to rule out or confirm a diagnosis of glomerulonephritis. Other ancillary staining might be necessary as for native kidney biopsies to establish specific recurrent or de novo kidney diseases (eg, Congo red stain).

Serological testing for donor-specific-antibodies should be performed as described in respective consensus documents.(*13*) Ancillary molecular tests, based on tissue and body fluids are emerging.

Preimplantation biopsies should be obtained, processed and reported as described by the Banff Working Group on Preimplantation Biopsies.(*14*)

# Banff Lesion Scores

Banff Lesion Scores assess the presence and the degree of histopathological changes in the different compartments of renal transplant biopsies, focusing primarily but not exclusively on the diagnostic features seen in rejection. These Banff Lesion Scores are not by themselves sufficient to reach the various Banff Diagnostic Categories in Table 1; the Additional Diagnostic Parameters - histopathological, molecular, serological and/or clinical - may be required to determine the diagnosis. For each Banff Lesion Score we give the current consensus definitions below. As new knowledge emerges, these might be refined for the forthcoming Banff 2019 update. A synopsis of their semi quantitative thresholds is given in Table 2. However, use of this threshold table without knowledge of the precise definitions and regulatory statutes underlying each Banff Lesion Score is strongly discouraged.

## Banff Lesion Score *i* (Interstitial Inflammation)

This score evaluates the degree of inflammation in nonscarred areas of cortex, which is often a marker of Acute T cell-Mediated Rejection (TCMR). As per the Banff update from 1997, areas that must not be considered for Banff Lesion Score *i* are “fibrotic areas, the immediate subcapsular cortex, and the adventitia around large veins and lymphatics”.(*5*) As can indirectly be derived from the definition of Banff Lesion Score *ti* in the 2007 update of the Banff classification, nodular infiltrates, if in unscarred cortex, are also considered for Banff Lesion Score *i*.(8) An asterisk shall be added to Banff Lesion Score i (eg, *i1**), “if there are more than 5-10% of eosinophils, neutrophils or plasma cells”.(*5*) Exemplary lesions are shown in Figure 2.

***i0*** - No inflammation or in less than 10% of unscarred cortical parenchyma

***i1*** - Inflammation in 10 to 25% of unscarred cortical parenchyma

***i2*** - Inflammation in 26 to 50% of unscarred cortical parenchyma

***i3*** - Inflammation in more than 50% of unscarred cortical parenchyma(*11*)

## Banff Lesion Score *t* (Tubulitis)

This Banff Lesion Score evaluates the degree of inflammation within the epithelium of the cortical tubules. As per the Banff 2003 update “Tubulitis—the presence of mononuclear cells in the basolateral aspect of the renal tubule epithelium” is one of the defining lesion of TCMR in kidney transplants.(*6*) According to Banff 1997, in tubules cut longitudinally, the score shall be determined as the number of mononuclear cells per 10 tubular epithelial cells, which is the average number of epithelial cells per tubular cross-section (Figure 3). Tubulitis must be present in at least 2 foci. We have emphasised this by rephrasing the criteria for Banff Lesion Score *t0* below; the most severely affected tubule determines the score.(*5, 11*) Please note also that we have returned from the altered definition with “leukocytes” in the Banff 2015 update (*11*) to “mononuclear cells” as given in the 1997 update.(*5*) According to the most recent Banff update from 2017, for Acute TCMR Grade IA, IB and Chronic Active TCMR Grade IA and IB but not Borderline (Banff Diagnostic Category 3), tubulitis is considered in all but severely atrophic cortical tubules. Tubulitis in severely atrophic tubules does not count towards a diagnosis of either Borderline, Acute or Chronic Active TCMR, and severely atrophic tubules are defined as diameter <25% of that of unaffected or minimally affected tubules on the biopsy, often with an undifferentiated-appearing, cuboidal or flattened epithelium (or in some cases even loss of epithelium with denudation of the tubular basement membrane), and pronounced wrinkling and/or thickening of the tubular basement membrane. This definition of severely atrophic tubules also includes very small, endocrine-like tubules with very narrow lumens, although the basement membranes of the latter may not be thickened.(*12*) An example of tubulitis in various stages of tubular atrophy is shown in Figure 4.

***t0*** - No mononuclear cells in tubules or single focus of tubulitis only

***t1*** - Foci with 1 to 4 mononuclear cells/tubular cross section (or 10 tubular cells)

***t2*** - Foci with 5 to 10 mononuclear cells/tubular cross section (or 10 tubular cells)

***t3*** - Foci with >10 mononuclear cells/tubular cross section or the presence of ≥2 areas of tubular basement membrane destruction accompanied by *i2*/*i3* inflammation and *t2* elsewhere(*12*)

## Banff Lesion Score v (Intimal Arteritis)

This Banff Lesion Score evaluates the presence and the degree of inflammation within the arterial intima. Arteries are defined as having at least 2 layers of smooth muscle cells in the media (Glossary of Terms, SDC, http://links.lww.com/TP/B604)***.*** Note that intimal arteritis (also referred to as endothelialitis and endarteritis) is defined by the presence of inflammatory cells, mainly lymphocytes and monocytes, in the subendothelial space of 1 or more arteries.(*10*) One such cell suffices. Examples of this lesion are shown in Figure 5. Intimal arteritis is a feature seen in both Acute TCMR and Active ABMR. For Banff Lesion Score *v* the most severely affected artery dictates the score.(*5*) Similar lesions in arterioles are only coded as an asterisk behind the Banff Lesion Score ah and are disregarded for Banff Lesion Score *v*. Infiltrates buried deeper in the intima are not considered for the *v* Banff Lesion Score but have been recognised as Chronic Active TCMR since the 2005 update,(*12*) and graded in 2017 update as Grade II.(*12*) In the presence of tubulointerstitial hemorrhage (see Glossary of Terms, SDC, http://links.lww.com/TP/B604) and/or and infarct (see Glossary of Terms, SDC, http://links.lww.com/TP/B604) an asterisk “*” is attached to the Banff Lesion Score *v* (eg, Banff *v0**, *v2**).(*5*)

***v0*** - No arteritis

***v1*** - Mild to moderate intimal arteritis in at least 1 arterial cross section

***v2*** - Severe intimal arteritis with at least 25% luminal area lost in at least 1 arterial cross section

***v3*** - Transmural arteritis and/or arterial fibrinoid change and medial smooth muscle necrosis with lymphocytic infiltrate in vessel(*11*)

## Banff Lesion Score *g* (Glomerulitis)

This Banff Lesion Score evaluates the degree of inflammation within glomeruli (Figure 6). Glomerulitis is a form of microvascular inflammation (MVI) and is a feature of activity and antibody interaction with tissue in ABMR. It can also be seen in recurrent or de novo glomerulonephritis which must be excluded by appropriate immunostains and EM.

Banff Lesion Score *g* is determined by the proportion of glomeruli showing glomerulitis defined as “complete or partial occlusion of ≥1 glomerular capillary by leukocyte infiltration and endothelial cell enlargement“.(*10*) Leukocytes include polymorphonuclear cells and mononuclear cells. Both endothelial cell enlargement and leukocyte(s) must contribute to the complete or partial occlusion. The denominator in this proportion is the number of nonsclerosed glomeruli in the biopsy.

***g0*** - No glomerulitis

***g1*** - Segmental or global glomerulitis in less than 25% of glomeruli

***g2*** - Segmental or global glomerulitis in 25 to 75% of glomeruli

***g3*** - Segmental or global glomerulitis in more than 75% of glomeruli(*11*)

## Banff Lesion Score *ptc* (Peritubular Capillaritis)

This Banff Lesion Score evaluates the degree of inflammation within peritubular capillaries (PTCs). Together with glomerulitis, peritubular capillaritis constitutes MVI as a feature of Active ABMR or Chronic Active ABMR. Peritubular capillaritis can be observed with pure Acute TCMR as well.

According to the Banff 2005 update, the Banff Lesion Score *ptc* is determined by the most severely involved PTC (Figure 7). PTCs are by definition found in the cortex, their medullary equivalent are medullary vasa recta. The number of luminal inflammatory cells includes polymorphonuclear and mononuclear leukocytes, with an asterisk “∗” used to indicate only mononuclear cells and absence of neutrophils. The extent of the PTC inflammation in the biopsy should be documented, either as focal (10-50% of cortical area) or diffuse (>50% of cortical area), but this does not contribute to the score. The presence of associated PTC dilatation may also be noted. Areas affected by acute pyelonephritis or necrosis and subcapsular cortex with nonspecific inflammation should not be scored. Inflammatory cells within PTCs must be distinguished from interstitial inflammation by careful examination of basement membrane stains (periodic acid-Schiff, silver). Inflammatory cells within veins and medullary capillaries (*vasa recta*) should not be scored.(*7*) Consequently, peritubular capillaritis and Banff Lesion Score *ptc* can only be assessed in the cortex after exclusion of areas of pyelonephritis and infarcted areas and exclusion of areas close to lymphoid aggregates to avoid confusion with lymphatic vessels. Banff Lesion Score *ptc* should not be based on longitudinally cut PTCs.(*8*) PTCs in areas affected by tubular atrophy and interstitial fibrosis must explicitly be considered for this Banff Lesion Score. Note that we have simplified the definition of *ptc0* from the original version in the Banff 2017 update.(*12*)

***ptc0*** - maximum number of leukocytes <3.

***ptc1*** - at least 1 leukocyte cell in >10% of cortical PTCs with 3–4 leukocytes in most severely involved PTC.

***ptc2*** - at least 1 leukocyte in >10% of cortical PTC with 5-10 leukocytes in most severely involved PTC

***ptc3*** - at least 1 leukocyte in >10% of cortical PTC with >10 leukocytes in most severely involved PTC(*11*)

## Banff Lesion Score *C4d*

This score evaluates the extent of staining for C4d on endothelial cells of PTCs and medullary *vasa recta* by immunofluorescence (IF) on snap frozen sections of fresh tissue or immunohistochemistry (IHC) on formalin-fixated and paraffin-embedded tissue. Although Banff 2008 states that areas of tubular atrophy and interstitial fibrosis have reduced PTC density that could affect the extent of staining,(*15*) scoring of C4d in such cortical areas is not excluded.(*8*) Scoring of C4d staining is based on the percentage of peritubular capillaries and vasa recta that has a linear, circumferential staining pattern (Figure 8). The minimal sample for evaluation is 5 high-power fields of cortex and/or medulla without scarring or infarction. *C4d* must not be scored in areas of infarction. On IF, staining should be at least 1+ in intensity.(*8*) Strong staining is not required for a positive reading for IHC.(*11*) In terms of extent of staining, with IF, Banff Lesion Score *C4d≥2* is considered positive and a criterion for antibody interaction with tissue and as equivalent to DSA (see Table 1 and SDC, Glossary of Terms, http://links.lww.com/TP/B604), whereas with IHC, Banff Lesion Score *C4d≥1* is counted as positive already.(*11*) Note that the definition below deviates from the one provided in the Banff 2015 update,(*11*) in that it explicitly allows scoring in medullary vasa recta as originally intended, not only PTCs. The thresholds remain unchanged.

***C4d0*** - No staining of PTC and medullary vasa recta (0%)

***C4d1*** - Minimal C4d staining (>0 but <10% of PTC and medullary vasa recta)

***C4d2*** - Focal C4d staining (10-50% of PTC and medullary vasa recta)

***C4d3*** - Diffuse C4d staining (>50% of PTC and medullary vasa recta)

## Banff Lesion Score *ci* (Interstitial Fibrosis)

This lesion score evaluates the extent of cortical fibrosis. The Banff Classification has never given a precise definition for individual areas of interstitial fibrosis (Figure 9). The reason for this is that Banff Lesion Score *ci* was meant to purely reflect the cortex composed of fibrous tissue, which does not necessarily correspond to areas that a pathologist would pick up as a patch of pathological tubulointerstitial fibrosis. The fraction of fibrous tissue in the cortex was considered as up to 5% for normal kidneys, hence the difference in cut-offs between *ci1* and *ct1*. A Working Group on this topic has produced useful reference guides (Figures 10 and 11).(*16*)

***ci0*** - Interstitial fibrosis in up to 5% of cortical area

***ci1*** - Interstitial fibrosis in 6 to 25% of cortical area (mild interstitial fibrosis)

***ci2*** - Interstitial fibrosis in 26 to 50% of cortical area (moderate interstitial fibrosis)

***ci3*** - Interstitial fibrosis in >50% of cortical area (severe interstitial fibrosis)(*11*)

##

## Banff Lesion Score *ct* (Tubular Atrophy)

This Banff Lesion Score evaluates the extent of cortical tubular atrophy which is usually tightly associated with the areas affected with interstitial fibrosis (Figure 9). Both correlate with time post transplantation in the setting of progressive disease of any cause. Accordingly, neither Banff Lesion Scores *ct* nor *ci* have diagnostic specificity, but both have significant correlation with allograft function and prognosis.

Historically the Banff classification has defined tubular atrophy as reflected in the Banff Lesion Score *ct* in the 1995 update(*4*) as tubules with a thickened basement membrane or a reduction of greater than 50% in tubular diameter. Banff Lesion Score *ct* is still based on this definition of tubular atrophy. The definitions of moderate and severe atrophy from the Banff 2017 update are irrelevant for Banff Lesion Score *ct*. In the following definition we have omitted the designation as “mild” for *ct1*, “moderate” for *ct2* and “severe” for *ct3* which was still included in the Banff 2015 update in order to avoid confusion between the definition of atrophy for an individual tubule as described above and the extent of tubular atrophy reflected in the Banff Lesion Score *ct*.

***ct0*** - No tubular atrophy

***ct1*** - Tubular atrophy involving up to 25% of the area of cortical tubules

***ct2*** - Tubular atrophy involving 26 to 50% of the area of cortical tubules

***ct3*** - Tubular atrophy involving in >50% of the area of cortical tubules(*11*)

## Banff Lesion Score *cv* (Vascular Fibrous Intimal Thickening)

This Banff Lesion Score reflects the extent of arterial intimal thickening in the most severely affected artery (see Definition of Terms, SDC, http://links.lww.com/TP/B604), not the average of all arteries.(*5*) It does not discriminate between bland arterial intimal fibrosis and fibrosis containing leukocytes (Figure 12), although the latter is more likely to reflect chronic rejection (ABMR and/or Chronic Active TCMR Grade II).(*12*) A visual analogue scale for application in daily practice is provided in Figure 13.

***cv0*** - No chronic vascular changes

***cv1*** - Vascular narrowing of up to 25% luminal area by fibrointimal thickening

***cv2*** - Vascular narrowing of 26 to 50% luminal area by fibrointimal thickening

***cv3*** - Vascular narrowing of more than 50% luminal area by fibrointimal thickening(*11*)

## Banff *cg* Score (Glomerular Basement Membrane Double Contours)

Banff Lesion Score *cg* is based on the presence and extent of glomerular basement membrane double contours or multilamination in the most severely affected glomerulus (Figure 14). Scoring should be carried out on periodic acid-Schiff (PAS) or silver stains; a designation as *cg1a* requires transmission EM to exclude *cg0*. With Banff Lesion Score *cg>0* (including both *cg1a* and *cg1b*) a diagnosis of transplant glomerulopathy (TG) (see Glossary of Terms, SDC, http://links.lww.com/TP/B604) can be made, if other causes can be excluded. Banff Lesion Score *cg>0* can be a feature of Chronic ABMR or Chronic Active ABMR, but can also be seen in association with thrombotic microangiopathy of other causes than ABMR, hepatitis C virus infection,(*17*) hypertensive glomerulopathy,(*18*) and glomerulonephritis. In analogy to Banff Lesion Score *g*, even in the presence of an explanation other than rejection for glomerular basement membrane double contours, Banff Lesion Score cg shall still be applied. Banff Lesion Score *cg* is not scored in ischemic or segmentally sclerosed glomeruli.(*1, 11*) Late Ischemic glomerulopathy is defined as “thickening, wrinkling and collapse of glomerular capillary walls associated with extracapillary fibrotic material”.(*1*) As stated above, the earliest lesion of TG (*cg1a*) requires transmission electron microscopy (EM) for diagnosis. In order to detect such lesions, it is recommended that at centers with EM capability, “ultrastructural studies should be performed in all biopsies from patients who are sensitized, have documented donor-specific antibody (DSA) at any time posttransplantation and/or who have had a prior biopsy showing C4d staining, glomerulitis and/or peritubular capillaritis”. It is also advised that EM be considered in all biopsies performed from 6 months posttransplantation onwards and in for-cause biopsies done from 3 months posttransplantation onwards to determine if early changes of TG are present, prompting testing for DSA“.(*10*) EM is also recommended for any biopsy done for the indication of increasing or new onset proteinuria.

***cg0*** - No glomerular basement membrane (GBM) double contours by light microscopy or electron microscopy (EM)

***cg1a*** - No GBM double contours by light microscopy but GBM double contours (incomplete or circumferential) in at least 3 glomerular capillaries by EM, with associated endothelial swelling and/or subendothelial electron-lucent widening

***cg1b*** - Double contours of the GBM in 1-25% of capillary loops in the most affected nonsclerotic glomerulus by light microscopy; EM confirmation is recommended if EM is available

***cg2*** - Double contours affecting 26 to 50% of peripheral capillary loops in the most affected –glomerulus

***cg3*** - Double contours affecting more than 50% of peripheral capillary loops in the most affected-glomerulus(*11*)

## Banff Lesion Score *mm* (Mesangial Matrix Expansion)

This score evaluates the percentage of glomeruli with “moderate mesangial matrix expansion” in relation to all nonsclerosed glomeruli. Banff 1997 defines moderate mesangial matrix increase as “expansion of the matrix in the mesangial interspace to exceed the width of 2 mesangial cells in the average in at least 2 glomerular lobules”.(*5*) An example is shown in Figure 15. Banff Lesion Score *mm* is currently not used to reach a diagnostic category and is purely descriptive.

***mm0*** - No more than mild mesangial matrix increase in any glomerulus

***mm1*** - At least moderate mesangial matrix increase in up to 25% of nonsclerotic glomeruli

***mm2*** - At least moderate mesangial matrix increase in 26-50% of nonsclerotic glomeruli

***mm3*** - At least moderate mesangial matrix increase in >50% of nonsclerotic glomeruli(*11*)

## Banff Lesion Score *ah* (Arteriolar Hyalinosis)

This score evaluates the extent of arteriolar hyalinosis (Figure 16). The first edition of the Banff Classification defined *ah* as “nodular hyaline afferent arteriolar thickening suggestive of cyclosporine toxicity”, however in Banff 1997 and later updates Banff Lesion Score *ah* is defined simply as PAS-positive arteriolar hyaline thickening, as a finding of “uncertain significance”. An asterisk “*” is added to the *ah* score when arteriolitis is present (eg, *ah0**, *ah2**).(*5*) Banff Lesion Score *ah* is currently not used to reach a diagnostic category and is purely descriptive.

***ah0*** - No PAS (periodic acid-Schiff)-positive hyaline arteriolar thickening

***ah1*** - Mild to moderate PAS-positive hyaline thickening in at least 1 arteriole

***ah2*** - Moderate to severe PAS-positive hyaline thickening in more than 1 arteriole

***ah3*** - Severe PAS-positive hyaline thickening in many arterioles(*11*)

## Banff Lesion Score *aah* (Hyaline Arteriolar Thickening)

This Banff Lesion Score provides an alternative way of quantifying arteriolar hyalinosis. It was proposed in the 2007 update, because of the insufficient reproducibility of the Banff Lesion Score *ah*.(*8*) This alternative tries to reach better reproducibility by focusing on circumferential or noncircumferential hyalinosis and the number of involved arterioles. Still, this lesion cannot be considered specific, ie, diagnostic for calcineurin inhibitor-related arteriolopathy. The use of this Banff Lesion Score *aah* has been left as optional since its introduction in 2007, no final decision has been reached whether it shall replace Banff Lesion Score *ah*. Banff Lesion Score *aah* is currently not used to reach a diagnostic category and is purely descriptive.

***aah0*** -  No typical lesions of calcineurin inhibitor-related arteriolopathy

***aah1*** -  Replacement of degenerated smooth muscle cells by hyaline deposits in only 1 arteriole, without circumferential involvement

***aah2*** - Replacement of degenerated smooth muscle cells by hyaline deposits in more than 1 arteriole, without circumferential involvement

***aah3*** - Replacement of degenerated smooth muscle cells by hyaline deposits with circumferential involvement, independent of the number of arterioles involved(*11*)

## Banff Lesion Score *ti* (Total Inflammation)

This lesion score evaluates the extent of total cortical inflammation. According to the Banff 2007 update and in contrast to the Banff Lesion Score *i*, all of the cortical parenchyma, including areas of interstitial fibrosis and tubular atrophy (IFTA), subcapsular cortex and perivascular cortex including nodular infiltrates are considered for ti scoring.(*8*) Mengel et al found Banff Lesion Score *ti* to be better predictive of poor graft outcomes than the Banff Lesion Score i in cases where at least mild IFTA was present.(*19*) The association between interstitial inflammation in areas of IFTA as reflected in Banff Lesion Score *i-IFTA* and decreased graft survival was noted by Mannon et al(*20*) and subsequently confirmed by others.(*21, 22*) As a consequence, Banff Lesion Score *ti* became part of the criteria for a diagnosis of Chronic Active TCMR Grade IA and IB;(*12*) Both Banff Lesion Scores *ti* and *i-IFTA* must be at least 2 to consider a diagnosis of Chronic Active TCMR Grade IA or IB.(*12*)

***ti0*** - No or trivial interstitial inflammation (<10% of total cortical parenchyma)

***ti1*** - 10–25% of total cortical parenchyma inflamed

***ti2*** - 26–50% of total cortical parenchyma inflamed

***ti3*** - >50% of total cortical parenchyma inflamed(*11*)

## Banff Lesion Score *i-IFTA* (Inflammation in Area of Interstitial Fibrosis and Tubular Atrophy)

This score evaluates the extent of inflammation in scarred cortex, ie, areas that qualify for Banff Lesion Scores *ci* and *ct* (Figure 17). The Banff Lesion Score *i-IFTA* was first introduced to the Banff Classification in 2015.(*11*) Both Banff Lesion Scores *ti* and *i-IFTA* must be at least 2 to consider a diagnosis of Chronic Active TCMR Grade IA or IB.(*12*)

***i-IFTA0*** - No inflammation or less than 10% of scarred cortical parenchyma

***i-IFTA1*** - inflammation in 10–25% of scarred cortical parenchyma

***i-IFTA2*** - inflammation in 26–50% of scarred cortical parenchyma

***i-IFTA3*** - inflammation in >50% of scarred cortical parenchyma(*11*)

# Banff Diagnostic Categories

Table 1 presents the Banff Diagnostic Categories and is based on the original table of the most recent Banff update from 2017(*12*). Readers should stay alert to future updates on the Banff Foundation website ([www.banfffoundation.org)](http://www.banfffoundation.org)) informed by updates to the Banff Classification from 2019 onwards.

# Critical Appraisal

Since 1991, the Banff classification has undergone several amendments, reflecting the growing body of knowledge in transplant pathology. These amendments have been based on a consensus reached at the biannual Banff meetings. This constant refinement based on emerging data is a strength of the Banff process and has led to the worldwide dominance of the Banff Classification for diagnostic practice, research and clinical trials. However, the iterative fashion in which the definitions and rules were published has dispersed the relevant content and created ambiguities. This has led to the creation of the Banff Rules and Dissemination Working Group in the aftermath of the Banff Meeting in Barcelona in March 2017. The aim of the Working group is not to alter the content of the Banff Classification. Rather, it shall collate all relevant Banff content in a central repository under the auspices of the Banff Foundation for Allograft Pathology, with a single updatable content, similar to the Union for International Cancer Control’s TNM Classification.(14) Changes in the content of the Banff Classification must only be made through review of evidence and expert consensus at the Banff meetings or within the relevant other Working Groups. Like the collation of content above, the following critical appraisal is based on this mission and does not touch on the content of the Banff Classification itself.

While the Banff Lesion Scores required for a diagnosis of ABMR have recently undergone a partial overhaul(*10*) and while a dedicated Working Group is re-examining the Banff Lesion Scores for TCMR, no or little effort has been devoted to the Additional Diagnostic Parameters in Table 3. For example, “Acute Tubular Injury In The Absence Of Any Other Cause” as a criterion for Active ABMR is as important as Banff Lesion Scores *v*, *g* or *ptc*,(*12*) yet this feature is still imperfectly defined, the last definition dating back to the 1995 update.(*4*) Another example is “infection”, which precludes the use of Banff Lesion Score *ptc* alone as a criterion for ABMR.(*11*) Use of the isolated term “infection” is ambiguous in the context of whether inflammation in the transplant should be considered as evidence for rejection or not. We would recommend treating these Additional Diagnostic Parameters like the Banff Lesion Scores, presenting them in clear and consistent wording, and, whenever necessary, by providing guidance through meaningful definitions elaborated over time through Working Groups and in alignment with the respective diagnostic criteria applied.

Among the Banff Lesion Scores, the Banff Lesion Score *cv* has a confusing array of terminologies, appearances and diagnostic implications. “Arterial fibrointimal thickening” or “vascular fibrous intimal thickening” imply a chronic fibrous change, whereas arterial intimal thickening can be cellular and nonfibrous in “transplant vasculopathy” or “chronic allograft arteriopathy”. As a manifestation of chronic TCMR it is defined as “arterial intimal fibrosis with mononuclear cell infiltration in fibrosis, formation of neointima”,(*12*) whereas, as a criterion for ABMR chronicity, it is defined as “arterial intimal fibrosis of new onset, excluding other causes; leukocytes within the sclerotic intima favour chronic ABMR if there is no prior history of biopsy-proven TCMR with arterial involvement but are not required”.(*12*) In clinical practice, it might not always be possible to exclude prior TCMR or to precisely diagnose “Arterial Intimal Fibrosis Of New Onset” as a criterion for ABMR chronicity.(*12*) A related problem is attached to Banff Lesion Score *cg*: “evidence of chronic TMA [thrombotic microangiopathy]” excludes the use of Banff Lesion Score *cg>0* as a criterion for ABMR chronicity, whereas Active ABMR can be diagnosed with TMA, as long as it is “in the absence of any other cause [than ABMR]” . Since Active ABMR causing TMA can lead to glomerular lesion qualifying as transplant glomerulopathy, it would make sense to change the cg criterion to only exclude chronic TMA of any other cause than ABMR.

The use of asterisks (“*”) attached to Banff Lesion Scores *v*, *i*, *ah* and *ptc*(*5, 7*) is problematic and widely neglected. Their reproducibility and diagnostic value are unknown, and they are ambiguous: an asterisk behind the Banff Lesion Score ptc signifies only mononuclear cells and absence of neutrophils, whereas the asterisk behind Banff Lesion Score *i* denotes a significant neutrophilic, eosinophilic or plasmacellular component in the infiltrate, and these different cell types can have widely differing implications. We suggest the Banff community should reassess these modifiers, either by improving their definitions and assigning them a significance or by abandoning them.

Inevitably, the Banff Classification has focused mainly on features of rejection, but with Banff Lesion Scores developed for other features with little or no guidance on their contribution to diagnosis. An example for this is Banff Lesion Score *aah*, originally intended to replace the poorly reproducible Banff lesion score *ah*.(*7*) However, its use is still optional and it has neither been widely adapted nor used in any of the Banff Diagnostic Categories. The Banff community should reassess arteriolar hyalinosis lesion scores, and clarify grading and diagnostic implications.

Regarding the Banff Diagnostic Categories, a clear diagnostic pathway should be recommended when dealing with Borderline or Acute TCMR (Banff Diagnostic Categories 3 and 4) in the presence of BK Virus Nephropathy, pyelonephritis or other infectious diseases of the transplant, as well as ABMR with glomerulitis in the presence of recurrent or de novo glomerulonephritis. These issues could be referred to the Banff TCMR and Glomerulonephritis Working Group respectively. The definition of Banff Borderline with regards to the Banff Lesion Score *i* threshold (*i0* or *i1*) is still ambiguous(*11*) but should be resolved by the TCMR Working Group.

There are uncertainties around the application of transmission electron microscopy in the diagnosis of ABMR which are currently being addressed by the Electron Microscopy Working Group. These issues include precise guidelines for indications and methods for application of EM in transplant biopsies; perhaps also the introduction of a new Banff Lesion Score for multilamination of the basement membranes of peritubular capillaries which we have covered as an Additional Diagnostic Parameter for now.

Another critical issue is related to the molecular diagnostics of ABMR and TCMR. While the current Banff classification endorses the use of molecular diagnostics in the definition of ABMR, there is limited guidance regarding methods and diagnostic cut-offs, which could be elaborated by the Molecular Working Group.

Lastly, the introduction of the new diagnostic categories of Chronic Active TCMR is likely to undergo changes informed by the TCMR Working Group. Prior to Banff 2017, there were no specific criteria for chronic active TCMR outside of arteries, and tubulitis was only scored in nonatrophic and mildly atrophic tubules, effectively excluding moderately and severely atrophic tubules. In order to avoid having 2 separate criteria for Banff Lesion Score *t* in Acute versus Chronic Active TCMR, it was decided that for both diagnoses tubulitis would be scored in all tubules except severely atrophic tubules.  The difference between Banff 2017 and previous versions of the classification with respect to Acute TCMR is that tubulitis in moderately atrophic tubules is now counted toward Banff Lesion Score *t*. As the latter was done for clarity and to avoid confusion rather than on the basis of specific evidence, it would be beneficial that future studies be done to address the most clinically relevant threshold for the level of atrophy permitted in scoreable tubules, especially for diagnosis of Acute TCMR. In addition, the 2017 changes to the TCMR criteria also suggest future work be aimed at examining the response of Chronic Active TCMR to steroids and other anti-T cell therapies (eg, thymoglobulin), determining if there are differences in this response between: 1) Grade IA vs IB Chronic Active TCMR; and 2) Biopsies with Chronic Active TCMR that would otherwise meet criteria for Acute TCMR (ie, with Banff Lesion Score *i≥2*) and those that would not (with Banff Lesion Score *i≤1*). The alignment of diagnoses from the spectrum of Acute TCMR with those from the spectrum of Chronic Active TCMR of different compartments could be problematic. For example, a biopsy with Banff Lesion Score *v1* fulfilling also the criteria for Chronic Active TCMR Grade IB would be diagnosed as the latter only,(*12*) as according to Banff 2017 a diagnosis of Chronic Active TCMR precludes the diagnosis even of higher grade Acute TCMR. In such cases, however, the use of modifying text independent from Banff diagnostic categories should be considered (eg, TCMR Grade II with a chronic active tubulointerstitial component; TCMR Grade II with isolated intimal arteritis [isolated v]).

# Prospects

Although this manuscript is intended to provide a comprehensive and convenient desk-top reference, it is destined to expire with the publication of the 2019 Banff update. After this update, a web resource will serve as the continuously updated go-to resource for the relevant Banff content. Depending on the progress in the definitions and diagnostic rule sets we are aiming to develop web-based resources such as diagnostic algorithms to further strengthen standardisation and reproducibility of the Banff Classification for clinical practice and research. It should be emphasised that the Banff Classification of Kidney Allograft Pathology does not cover all relevant aspects of transplantation medicine. Allograft transplantation only reaches 10% of patients needing new organs. Through regenerative medicine and tissue engineering and other optimizing initiatives we will eventually be able to provide organs to everyone in need. For this we will need a new Banff Classification of Tissue Engineering Pathology (TEP)(*23, 24*) reflecting the new challenges of delivering the right cells to the right places in a bioengineered organ and having them function normally. Rejection will no longer be the primary threat in bioengineered organs. For a decade or more the new Banff Classification of TEP will be used concurrently with the existing Banff Classification of Allograft Pathology.

Getting the right cells in the right places sounds simple but in fact we have poor knowledge of what all the normal cell types in transplanted organs are. For instance, in the kidney we have traditionally taught that there are 26 cell types(*25*) but in fact high throughput single cell analysis in the Human Cell Atlas Project (HCAP)(*26-28*) shows many more than that, and can determine not only cell identity, but also lineage, and activation state. The transplantation and transplantation pathology community need to embrace Human Cell Atlas technology so we are not blindsided by this new technology. The scale of the likely impact of the HCAP on nephrology and transplantation is currently being analysed (Moghe I, Magor B, and Solez K, manuscript in preparation, 2018).

# Acknowledgments

The authors would like to acknowledge the help in the preparation of the visual analogue scales from Christopher Bellamy, Alton “Brad” Farris and Daniel Serón.

# References

1. Solez K, Axelsen RA, Benediktsson H, et al. International standardization of criteria for the histologic diagnosis of renal allograft rejection: the Banff working classification of kidney transplant pathology. *Kidney Int*. 1993;44:411-422.

2. Mengel M, Sis B, Halloran PF. SWOT analysis of Banff: strengths, weaknesses, opportunities and threats of the international Banff consensus process and classification system for renal allograft pathology. *Am J Transplant*. 2007;7:2221-2226.

3. Becker JU, Chang A, Nickeleit V, Randhawa P, Roufosse C. Banff borderline changes suspicious for acute T-cell mediated rejection: where do we stand? *Am J Transplant*. 2016;16:2654-2660.

4. Solez K, Benediktsson H, Cavallo T, et al. Report of the Third Banff Conference on Allograft Pathology (July 20-24, 1995) on classification and lesion scoring in renal allograft pathology. *Transplant Proc*. 1996;28:441-444.

5. Racusen LC, Solez K, Colvin RB, et al. The Banff 97 working classification of renal allograft pathology. *Kidney Int*. 1999;55:713-723.

6. Racusen LC, Halloran PF, Solez K. Banff 2003 meeting report: new diagnostic insights and standards. *Am J Transplant*. 2004;4:1562-1566.

7. Solez K, Colvin RB, Racusen LC, et al. Banff '05 Meeting Report: differential diagnosis of chronic allograft injury and elimination of chronic allograft nephropathy ('CAN'). *Am J Transplant*. 2007;7:518-526.

8. Solez K, Colvin RB, Racusen LC, et al. Banff 07 classification of renal allograft pathology: updates and future directions. *Am J Transplant*. 2008;8:753-760.

9. Mengel M, Sis B, Haas M, et al. Banff 2011 Meeting Report: New Concepts in Antibody-Mediated Rejection. *Am J Transplant*. 2012;12:563-570.

10. Haas M, Sis B, Racusen LC, et al. Banff 2013 meeting report: inclusion of c4d-negative antibody-mediated rejection and antibody-associated arterial lesions. *Am J Transplant*. 2014;14:272-283.

11. Loupy A, Haas M, Solez K, et al. The Banff 2015 Kidney meeting report: Current challenges in rejection classification and prospects for adopting molecular pathology. *Am J Transplant*. 2017;17:28-41.

12. Haas M, Loupy A, Lefaucheur C, et al. The Banff 2017 Kidney Meeting Report: Revised Diagnostic Criteria for Chronic Active T Cell-Mediated Rejection, Antibody-Mediated Rejection, and Prospects for Integrative Endpoints for Next-Generation Clinical Trials. *Am J Transplant*. 2018;18:293-307.

13. Tait BD, Susal C, Gebel HM, et al. Consensus guidelines on the testing and clinical management issues associated with HLA and non-HLA antibodies in transplantation. *Transplantation*. 2013;95:19-47.

14. Liapis H, Gaut JP, Klein C, et al. Banff Histopathological Consensus Criteria for Preimplantation Kidney Biopsies. *Am J Transplant*. 2017;17:140-150.

15. Ishii Y, Sawada T, Kubota K, Fuchinoue S, Teraoka S, Shimizu A. Loss of peritubular capillaries in the development of chronic allograft nephropathy. *Transplant Proc*. 2005;37:981-983.

16. Farris AB, Chan S, Climenhaga J, et al. Banff fibrosis study: multicenter visual assessment and computerized analysis of interstitial fibrosis in kidney biopsies. *Am J Transplant*. 2014;14:897-907.

17. Baid-Agrawal S, Farris AB 3^rd^, Pascual M, et al. Overlapping pathways to transplant glomerulopathy: chronic humoral rejection, hepatitis C infection, and thrombotic microangiopathy. *Kidney Int*. 2011;80:879-885.

18. Olson JL. Renal Disease Caused by Hypertension. In: Jennette JC, Olsen SL, Silva FG, et al, eds. Heptinstall’s Pathology of the Kidney. 7th Edition ed. Wolters Kluwer: Philadelphia, PA, USA; 2015, 849-896.

19. Mueller TF, Einecke G, Reeve J, et al. Microarray analysis of rejection in human kidney transplants using pathogenesis-based transcript sets. *Am J Transplant*. 2007;7:2712-2722.

20. Mannon RB, Matas AJ, Grande J, et al. Inflammation in areas of tubular atrophy in kidney allograft biopsies: a potent predictor of allograft failure. *Am J Transplant*. 2010;10:2066-2073.

21. Lefaucheur C, Gosset C, Rabant M, et al. T cell-mediated rejection is a major determinant of inflammation in scarred areas in kidney allografts. *Am J Transplant*. 2018;18:377-390.

22. Nankivell BJ, Shingde M, Keung KL, et al. The causes, significance and consequences of inflammatory fibrosis in kidney transplantation: The Banff i-IFTA lesion. *Am J Transplant*. 2018;18:364-376.

23. Solez K, Fung KC, Saliba KA, et al. The bridge between transplantation and regenerative medicine: Beginning a new Banff classification of tissue engineering pathology. *Am J Transplant*. 2018;18:321-327.

24. Solez K. Kim Solez, Edmonton, Alberta, Canada Banff: A Unique Start Setting Standards for Consensus Conferences. *Transplantation.* 2017;101:2264-2266.

25. Al-Awqati Q, Oliver JA. Stem cells in the kidney. *Kidney Int*. 2002;61:387-395.

26. Stubbington MJT, Rozenblatt-Rosen O, Regev A, Teichmann SA. Single-cell transcriptomics to explore the immune system in health and disease. *Science*. 2017;358:58-63.

27. Rozenblatt-Rosen O, Stubbington MJT, Regev A, Teichmann SA. The Human Cell Atlas: from vision to reality. *Nature*. 2017;550:451-453.

28. Regev A, Teichmann SA, Lander ES, et al. The Human Cell Atlas. *Elife*. 2017;6. doi:10.7554/eLife.27041.

29. Farris AB, Adams CD, Brousaides N, et al. Morphometric and visual evaluation of fibrosis in renal biopsies. *J Am Soc Nephrol.* 2011;22:176-186.

# Tables

## **Table 1:**

Banff Diagnostic Categories form the core of the Banff Classification of Renal Allograft Pathology. We refer to the Banff Lesion Scores in the main body of this review as well as to the Additional Diagnostic Parameters listed in Table 3. Note that diagnoses from various Banff Diagnostic Categories can coexist in a given biopsy, eg, Acute TCMR Grade IB, Chronic Active ABMR, moderate Interstitial Fibrosis and Tubular Atrophy and Calcineurin Inhibitor Toxicity. From each Banff Diagnostic Category except for 6, only 1 diagnosis must be made. Note that the Banff Diagnostic Categories Suspicious for Acute/Active ABMR, Suspicious for Chronic ABMR, Suspicious for Chronic Active ABMR from the Banff 2015 update have been deleted.(*12*)

Remark: This Table should be brought in landscape format.

| **Category 1: Normal biopsy or nonspecific changes** | |
| --- | --- |
| Requires exclusion of any diagnosis form the Banff Diagnostic Categories 1-4, 6 below. | |
| **Category 2: Antibody-mediated changes**  Use the Diagnostic Criteria Groups (right column) to reach 1 Diagnosis (left column) | |
| **Diagnoses** | **Diagnostic Criteria Groups** |
| **C4d Staining Without Evidence Of Rejection** Banff Lesion Score *C4d>1* (immunofluorescence on fresh frozen tissue) OR *C4d>0* (immunohistochemistry on paraffin-embedded tissue)  AND  Banff Lesion Scores *t0*, *v0*, no Arterial Intimal Fibrosis With Mononuclear Cell Inflammation In Fibrosis And Formation Of Neointima, no criterion from Group 1 (ABMR activity), no criterion from Group 4 (histologic features of ABMR chronicity), no Increased Expression Of Thoroughly Validated Gene Transcripts/Classifiers In The Biopsy Tissue Strongly Associated With ABMR | **Criteria Group 1** **ABMR activity:** - Banff Lesion Score *g>0* in the absence of glomerulonephritis and/or Banff Lesion Score *ptc>0* in the absence of TCMR or Borderline - Banff Lesion Score *v>0 -* Acute thrombotic microangiopathy in the absence of any other cause (Figure 18) - Acute tubular injury in the absence of any other apparent cause |
| **Active Antibody-Mediated rejection (ABMR)** No criterion of ABMR chronicity (Criteria Group 4)  AND  At least on criterion from Criteria Group 1 (ABMR activity)  AND  At least 1 criterion from Criteria Group 2 (antibody interaction with tissue)  AND  At least 1 criterion from Criteria Group 3 (DSA or equivalents) | **Criteria Group 2** **Antibody interaction with tissue:** - Banff Lesion Score *C4d>1* (IF on fresh frozen tissue) or *C4d>0* (IHC on paraffin-embedded tissue)- At least moderate microvascular inflammation (*g+ptc>1*) in the absence of Borderline (Diagnostic Category 3) or acute T cell-mediated rejection (TCMR; Diagnostic Category 4). If Borderline or acute TCMR are present, (Banff Lesion Scores *g+ptc)>1* is not sufficient and Banff Lesion Score *g>1* is required. - Increased expression of thoroughly validated gene transcripts/classifiers in the biopsy tissue strongly associated with ABMR |
| **Chronic Active ABMR**  At least 1 feature of ABMR chronicity (Criteria Group 4)  AND  At least 1 criterion of antibody interaction with tissue (Criteria Group 2)  AND  At least 1 criterion of DSA or equivalents (Criteria Group 3) | **Criteria Group 3 Donor-specific antibody or equivalents:** - Donor-specific antibody (anti-HLA or other specificity) - Banff Lesion Score *C4d>1* (IF on fresh frozen tissue) or *C4d>0* (IHC on paraffin-embedded tissue) - Increased expression of thoroughly validated gene transcripts/classifiers in the biopsy tissue strongly associated with ABMR |
| **Chronic ABMR**  Banff 2017 permits the use of this term for biopsy specimens showing TG and/or peritubular capillary basement membrane multilayering in the absence of criterion of current/recent antibody interaction with the endothelium (Criteria Group 2) but with a prior documented diagnosis of active or chronic active ABMR or documented prior evidence of DSA | **Criteria Group 4 Histologic features of ABMR chronicity** - Banff Lesion Score *cg>0* (by light microscopy or electron microscopy, if available), excluding biopsies with evidence of chronic thrombotic microangiopathy  - 7 or more layers in 1 cortical peritubular capillary and 5 or more in 2 additional capillaries, avoiding portions cut tangentially by electron microscopy, if available (Severe Peritubular Capillary Basement Membrane Multilayering); Figure 19) - Arterial intimal fibrosis of new onset, excluding other causes; leukocytes within the sclerotic intima favour chronic ABMR if there is no prior history of biopsy-proven TCMR with arterial involvement but are not required |
| **Category 3: Suspicious (Borderline) for Acute TCMR** | |
| Foci of Banff Lesion Score *t>0* AND Banff Lesions Score *i≤1* (retaining the Banff Lesion Score *i1* threshold from Banff 2005 is permitted but it must be made transparent in the methods section of reports and publications) OR Foci of Banff Lesion Score *t1* AND Banff Lesion Score *i≥2* | |
| **Category 4: TCMR** | |
| **Acute TCMR IA** Banff Lesion Score *i≥2* AND Banff Lesion Score *t2* | |
| **Acute TCMR IB** Banff Lesion Score *i≥2* AND Banff Lesion Score *t3* | |
| A**cute TCMR IIA** Banff Lesion Score *v1* regardless of Banff Lesion Scores *i* or *t* | |
| **Acute TCMR IIB** Banff Lesion Score *v2* regardless of Banff Lesion Scores *i* or *t* | |
| **Acute TCMR III** Banff Lesion Score *v3* regardless of Banff Lesion Scores *i* or *t* | |
| **Chronic Active TCMR Grade IA**  Banff Lesion Score *ti≥2*  AND  Banff Lesion Score *i-IFTA≥2*, other known causes of i-IFTA (eg, pyelonephritis, BK-virus nephritis etc.) ruled out  AND Banff Lesion Score *t2* | |
| **Chronic Active TCMR Grade IB**  Banff Lesion Score *ti≥2* AND  Banff Lesion Score *i-IFTA≥2*, other known causes of i-IFTA ruled out AND  Banff Lesion Score *t3* | |
| **Chronic Active TCMR Grade II**  Arterial intimal fibrosis with mononuclear cell inflammation in fibrosis and formation of neointima | |
| **Category 5: Interstitial Fibrosis and Tubular Atrophy** | |
| **Grade I (Mild)** Banff Lesion Score *ci1* OR Banff Lesion Score *ct1* | |
| **Grade II (Moderate)** Banff Lesion Score *ci2* OR Banff Lesion *ct2* | |
| **Grade III (Severe)** Banff Lesion Score *ci3* OR Banff Lesion Score *ct3* | |
| **Category 6: Other Changes not considered to be caused by Acute or Chronic Rejection (Figure 20)** | |
| BK-Virus Nephropathy Posttransplant Lymphoproliferative Disorder Calcineurin Inhibitor Toxicity Acute Tubular Injury Recurrent Disease De Novo Glomerulopathy (other than transplant glomerulopathy) Pyelonephritis Drug-Induced Interstitial Nephritis | |

## **Table 2:**

This is a synopsis of the thresholds for all Banff Lesion Scores. The user of this table should be familiar with the exact definitions underlying each individual Banff Lesion Score. Reliance on these thresholds alone without consideration of the regulatory statutes behind these scores is strongly discouraged. Abbreviations: EM: electron microscopy, LM: light microscopy, max.: maximum, PTC: peritubular capillary.

| Banff Lesion Score | Abbreviation | 0 | 1 | 2 | 3 |
| --- | --- | --- | --- | --- | --- |
| Interstitial Inflammation | *i* | <10% | 10-25% | 26-50% | >50 |
| Tubulitis | *t* | None | 1-4/tubular cross section or 10 tubular cells | 5-10 | >10 or foci of tubular basement membrane destruction with i≥2 and t2 elsewhere |
| Intimal Arteritis | *v* | None | <25% luminal area lost | ≥25% luminal area lost | Transmural and/or fibrinoid change and medial smooth muscle necrosis |
| Glomerulitis | *g* | None | <25% | 25-75% | >75% |
| Peritubular Capillaritis | *ptc* | <3 leukocytes/PTC | ≥1 leukocyte in ≥10% of PTCs with max. of 3-4/PTC | ≥1 leukocyte in ≥10% of PTCs with max. of 5-10/PTC | ≥1 leukocyte in ≥10% of PTCs with max. of >10/PTC |
| C4d | *C4d* | None | <10% | 10-50% | >50% |
| Interstitial Fibrosis | *ci* | ≤5% | 6-25% | 26-50% | >50% |
| Tubular Atrophy | *ct* | None | ≤25% | 26-50% | >50% |
| Vascular Fibrous Intimal Thickening | *cv* | None | ≤25% | 26-50% | >50% |
| Glomerular Basement Membrane Double Contours | *cg* | None | 1a: only by EM | 26-50% | >50% |
|  |  |  | 1b: ≤25% by LM |  |  |
| Mesangial Matrix Expansion | *mm* | None | ≤25% | 26-50% | >50% |
| Arteriolar Hyalinosis | *ah* | None | Mild to moderate in ≥1 | Moderate to severe in ≥1 | Severe in many |
| Hyaline Arteriolar Thickening | *aah* | None | 1 without circumferential | ≥1 without circumferential | circumferential |
| Total Inflammation | *ti* | <10% | 10-25% | 26-50% | >50% |
| Inflammation In Area of Interstitial Fibrosis and Tubular Atrophy | *i-IFTA* | <10% | 10-25% | 26-50% | >50% |

## **Table 3:**

These Additional Diagnostic Parameters, some histopathologic, some clinical, are derived from the diagnostic algorithms in Table 1. Depending on the constellation of findings they may be required in addition to the Banff Lesion Score to determine the Banff Diagnostic Categories.

| **Parameter** | **Required for Diagnostic Category** |
| --- | --- |
| Acute Thrombotic Microangiopathy In The Absence Of Any Other Cause (Figure 18) | Active and Chronic Active ABMR |
| Acute Tubular Injury In The Absence Of Any Other Apparent Cause | Active ABMR |
| Absence Of Recurrent Or De Novo Glomerulonephritis | Active and Chronic Active ABMR |
| Infection | Active and Chronic Active ABMR |
| Arterial Intimal Fibrosis Of New Onset, Excluding Other Causes | Chronic ABMR and Chronic Active ABMR |
| Leukocytes Within The Sclerotic [Arterial] Intima Favor Chronic ABMR | Chronic Active ABMR and Chronic ABMR |
| Increased Expression Of Thoroughly Validated Gene Transcripts/Classifiers In The Biopsy Tissue Strongly Associated With ABMR | C4d Staining Without Evidence Of Rejection, Active ABMR, Chronic Active ABMR, Chronic ABMR |
| Severe Peritubular Capillary Basement Membrane Multilayering (Figure 19) | Chronic ABMR and Chronic Active ABMR |
| Arterial Intimal Fibrosis With Mononuclear  Cell Inflammation In Fibrosis And Formation Of Neointima | Chronic Active TCMR Grade II |
| Prior evidence of DSA | Chronic ABMR |
| Serologic evidence of donor-specific  antibodies (DSA to HLA or other antigens). | Active ABMR, Chronic Active ABMR, Chronic ABMR |
| Prior Documented Diagnosis Of Active Or Chronic Active ABMR | Chronic ABMR |
| Prior History Of TCMR | Chronic Active ABMR and Chronic ABMR |
| Evidence Of Chronic TMA | Chronic Active ABMR and Chronic ABMR |
| C4d-Staining On Fresh-Frozen Or Paraffin-Embedded Tissue | C4d Staining Without Evidence Of Rejection, Active ABMR, Chronic Active ABMR, Chronic ABMR |
| BK-Virus Nephropathy | Other Changes Not Considered To Be Caused By Acute Or Chronic Rejection |
| Posttransplant Lymphoproliferative Disorder |  |
| Calcineurin Inhibitor Toxicity |  |
| Acute Tubular Injury |  |
| Recurrent Disease |  |

# Figure Legends

Figure 1:

The content of the Banff Classification of Kidney Allograft Pathology can be inventoried as Banff Lesion Scores and Additional Diagnostic Parameters required by the algorithms behind the Banff Diagnostic Categories to reach a diagnosis. Moreover, overarching Definitions are important and inform eg, how one or even several Banff Lesion Scores are applied. Abbreviation: TMA, thrombotic microangiopathy

Figure 2. Banff Lesion Score *i* (interstitial Inflammation in nonscarred areas of the cortex). A) Interstitial inflammation in nonscarred areas of the cortex. This Banff Lesion Score, often a marker of TCMR, ranges from 0 to 3, based on the percentage of nonscarred cortex involved, and is usually dominated by mononuclear cells in the case of Acute TCMR. Note the contrast between the noninfiltrated interstitium in the right half of the micrograph and the infiltrate in the oedema between the tubules on the left (long arrow). PAS, original magnification x400. B) An example of plasma cell rich interstitial inflammation. If the infiltrate comprises more than 5-10% of either eosinophils, neutrophils or plasma cells an asterisk is added to the Banff Lesion Score i (eg, *i1**). H&E (hematoxylin and eosin), original magnification x400.

Figure 3. Banff Lesion Score *t* (tubulitis) in nonatrophic or mildly atrophic tubules. These images display various degrees of tubulitis which is characterised by the presence of mononuclear cells on the basolateral aspect of the tubular epithelial cells, within the confines of the basement membrane. Mononuclear cells (long and short arrows) are noticeable by their characteristic halo and smaller nucleus and more condensed chromatin compared to tubular epithelial cells. A) Banff Lesion Score *t0* - Cortical tubules without tubulitis which would be scored as *t0*. H&E, Original magnification x200. B) Banff Lesion Score *t1* - defined as foci of 1-4 mononuclear cells (arrows) per tubular cross section or per 10 tubular epithelial cells. PAS, original magnification x400. C) Banff Lesion Score *t2* - defined as 5-10 mononuclear cells per tubular cross section or per 10 epithelial cells (long arrows). Note that the tubule to the left displays mild tubulitis (short arrows), but the most severely affected tubule dictates the score. PAS, original magnification x400. D) Banff Lesion Score *t3* - defined as foci with >10 mononuclear cells/tubular cross section. Note that for this particular tubule the denominator is per 10 tubular epithelial cells as this tubule is sectioned longitudinally. PAS, Original magnification x400.

Figure 4. Banff Lesion Score *t* (tubulitis) in moderately atrophic tubules. In biopsies with Banff Lesion Scores *i*, *ti* and *i-IFTA* sufficient for a diagnosis of Acute TCMR Grade IA, IB or Chronic Active TCMR Grade IA and IB, Banff Lesion Score *t* must also be scored in moderately atrophic cortical tubules. Moderately atrophic tubules are defined as having less than 50% down to 25% of the diameter of the surrounding “unaffected or minimally affected [cortical] tubules in the biopsy”.(*12*) This example shows such unaffected or minimally affected tubules with their diameter marked in black. Their mean diameter in this image would be around 48 µm. The tubule with the diameter marked in grey has a diameter of 27 µm which is more than 50% of 48 µm. Thus, this tubule would still qualify as mildly atrophic. It is heavily infiltrated with mononuclear cells (grey arrows). In contrast, the tubule with the diameter of 20 µm marked in red is moderately atrophic. The mononuclear tubulitis in this particular tubule must be scored towards Banff Lesion Score *t* in this biopsy which was diagnosed as Acute TCMR Grade IB. PAS, original magnification x400.

Figure 5. Banff Lesion Score v (intimal arteritis). These photomicrographs demonstrate intimal arteritis, characterised by the presence of inflammatory cells beneath the lining endothelial cells. A) Banff Lesion Score *v1* - Mild to moderate arteritis with mononuclear cells (long arrows) immediately beneath lifted endothelial cells (short arrow). H&E, original magnification x200. B) Banff Lesion Score *v2* - Severe intimal arteritis involving over 25% of the arterial lumen with mononuclear cells (long arrows) immediately beneath lifted endothelial cells (short arrow). H&E, original magnification x200; C) Banff Lesion Score *v3* -Transmural arteritis with fibrinoid necrosis in the media (long arrow) and mononuclear infiltrate in the arterial wall (short arrows). Intimal arteritis can be seen in both Acute TCMR Grade II and III and Active ABMR. The most severely affected artery determines the score. Masson trichrome, original magnification x100; D) This image demonstrates an area of Interstitial hemorrhage characterised by extravasation of red blood cells into the surrounding interstitium (arrow). Although there is not a specific Banff Lesion Score for this feature, it can be recorded by attaching an asterisk to the v score (eg, *v**). Not that this asterisk attached to Banff Lesion Score *v* is not specific for interstitial hemorrhage as an area of cortical infarct (not shown) would also be coded like this. H&E, original magnification x400.

Figure 6. Banff Lesion Score *g* (glomerulitis). Glomerulitis is a form of microvascular inflammation and a feature of ABMR activity. A) Segmental glomerulitis; PAS, original magnification x400. B) Global glomerulitis. Note the characteristic complete or partial occlusion of capillary loops by leukocytes (short arrows) and endothelial cell swelling (long arrows). The score of *g0* to *g3* is determined by the percentage of glomeruli involved with either segmental or global glomerulitis. Complete or partial occlusion of a single capillary loop suffices to mark the respective glomerulus as involved by the glomerulitis. PAS, original magnification x400.

Figure 7. Banff Lesion Score *ptc* (peritubular capillaritis). Peritubular capillaritis is a form of microvascular inflammation and a feature of ABMR activity. Each image demonstrates the various *ptc* scores which are in themselves determined by the number of inflammatory cells present within capillary lumina. A) Banff Lesion Score *ptc1* - Mild peritubular capillaritis defined as at least 1 cell in ≥ 10% of cortical PTCs (short arrows) with 3-4 in the most severely involved PTC (long arrow). Please note the slightly distended, open appearance of the capillary which can be a helpful feature; PAS, original magnification x400. B) Banff Lesion Score *ptc2* - Moderate peritubular capillaritis defined as at least 1 cell in ≥ 10% of cortical PTCs (short arrows) with 5-10 in most severely involved PTC (long arrow); PAS, original magnification x400. C) Banff Lesion Score *ptc3* - Severe peritubular capillaritis defined as at least 1 cell in ≥ 10% of cortical PTCs (short arrows) with >10 in most severely involved PTC (long arrow). PAS, original magnification x400. D) This peritubular capillary is cut longitudinally (short arrow) and although containing 4 mononuclear cells is to be disregarded for scoring. However, the neighbouring peritubular capillary (long arrow) is cut orthogonally and would qualify for Banff Lesion Score *ptc1* provided that at least 10% of all PTCs contain at least 1 leukocyte. PAS, original magnification x400.

Figure 8. Banff Lesion Score C4d. A) Immunohistochemical (IHC) staining with peroxidase yielding a brown reaction product for C4d. An example of *C4d3*, this image demonstrates linear and circumferential staining of endothelial cells in virtually all peritubular capillaries. The staining was similar in all areas of the cortex and the medulla. The proportion of stained peritubular capillaries and medullary vasa recta informs the score. B) Immunofluorescence (IF) staining for *C4d*. This image shows an example of a Banff Lesion Score of *C4d3*; using IF, a minimum score of *C4d≥2* is considered positive. In addition to this, the staining intensity for an individual capillary or medullary vas rectum must be at least 1+ on the usual scale from negative, trace, 1+, 2+ to 3+. Indirect IF, mouse antihuman C4d followed by fluorescein isothiocyanate-conjugated antimouse IgG, original magnification x100.

Figure 9. Banff Lesion Scores for *ct* (tubular atrophy) and *ci* (interstitial fibrosis). The *ci* and *ct* scores are both based on calculating the total percentage of cortex involved and require a diligent assessment of all foci of *ct* and *ci* as this process is often multifocal; *ct* and *ci* scores may not always be equally advanced. A) This image demonstrates an area of nonatrophic tubules (long arrow), compared to an area of tubular atrophy (short arrow) without an obvious increase in interstitial fibrosis. PAS, original magnification x200. There are different morphological types of tubular atrophy with differing histological appearances including conventional, thyroidisation and endocrine-like types; B) Tubular atrophy of conventional type with interstitial fibrosis. Tubular areas are separated by areas of interstitial fibrosis and tubules show thickened basement membranes and >50% reduction in tubular diameter (long arrows). PAS, original magnification x200. C) Thyroidisation type atrophy. Here tubules appear dilated, have flattened epithelial cells and contain eosinophilic and brightly periodic-acid-Schiff-positive uromodulin casts (long arrow). PAS, original magnification x200. D) endocrine-like type, characterised by shrunken tubules with cuboidal epithelium and ‘tubular simplification’ (long arrow). Compared to the other types of tubular atrophy, endocrine-like type does not have thickened basement membranes but still counts toward the ct score. PAS, original magnification x 400.

Figure 10. Visual analogue scales provided by the Banff Working Group on Fibrosis. This Working Group developed schematic diagrams to facilitate and standardise scoring of Banff Lesion Scores *ci* and *ct*. A). Scale for the assessment of interstitial fibrosis without tubular atrophy; B) Scale for the assessment of diffuse tubular atrophy with ‘replacement fibrosis’.(*29*) Reproduced with kind permission from American Journal of Transplantation.

Figure 11. More visual analogue scales provided by the Banff Working Group on Fibrosis. (16). A). Scale for the assessment of patchy (left) and confluent (right) interstitial fibrosis without glomeruli; B) Scale for patchy (left) and confluent (right) fibrosis with glomeruli.(*29*) Reproduced with kind permission from American Journal of Transplantation.

Figure 12. Banff Lesion Score *cv* (vascular fibrous intimal thickening). A) Banff Lesion Score *cv1* – Very mild purely fibrous thickening of the arterial intima (arrow). PAS, original magnification x200. B) Purely fibrous intimal thickening is depicted here in between the arrows in a trichrome stain. Note that this type of fibrous intimal thickening can also represent chronic damage in ABMR. Masson’s trichrome, original magnification x400. C) Arterial fibrous intimal thickening in between the arrows. Note the multiplication of the internal elastic lamina. Trichrome-elastica, original magnification x400. D) Severe fibrointimal thickening *cv3*, with mononuclear infiltrates (long arrow) and foam cells (short arrow) in the fibrotic intima which can be a feature of both Chronic Active TCMR and Chronic Active or Chronic ABMR. Both types of lesion qualify for Banff Lesion Score *cv*, the score is determined by the loss of luminal area as shown in Figure 12 below. H&E, original magnification x100.

Figure 13. Visual analogue scale for the determination of Banff Lesion Score *cv* (Arterial fibrous intimal thickening). The remaining luminal area is related to the square of the remaining luminal radius. Thus, relatively modest decreases in luminal radius of 13% or 29% translate into relatively large reductions in luminal area of 25% or 50%, reflecting the thresholds for Banff Lesion Score *cv*.

Figure 14. Banff Lesion Score *cg* (glomerular basement membrane double contours). This score represents the presence and extent of glomerular basement membrane double contours, a criterion for Chronic Active ABMR. The score ranges from 0 to 3 and is based on the percentage of capillary loops with double contours as evident on EM (Banff Lesion Score *cg1a*) or LM (*cg1b* to *cg3*) in the most severely affected glomerulus. A) *cg1a* - GBM with double contours (black arrows point to areas of original basement membrane and red arrows point to areas of new basement membrane formation), visible by electron microscopy only. Double contours such as those noted in this image must be accompanied by endothelial cell swelling and/or subendothelial rarefaction, and must involve at least 3 glomerular capillaries by EM for a score of *cg1a*. Scores of greater than *cg1a* are based on light microscopic appearance which can best be examined by silver stains. Transmission EM, original magnification x8,000. B) Banff Lesion Score *cg1b* - Double contours (arrow) identified on LM which involve up to 25% of the capillary loops of this most affected glomerulus. Jones silver stain, original magnification x400. C) Banff Lesion Score *cg2* - Double contours (arrows) present in 26-50% of this most affected glomerulus; Jones silver stain, original magnification x400. D) Banff Lesion Score *cg3* - Double contours (arrows) present in >50% of this most affected glomerulus. Jones silver stain, original magnification x 400.

Figure 15. Banff Lesion Score *mm* (mesangial matrix expansion). This glomerulus fulfils the criteria for moderate mesangial matrix expansion with more than 2 mesangial cells in these 2 adjacent glomerular lobules (arrows). The proportion of glomeruli with such mesangial matrix expansion among all nonsclerosed glomeruli informs the score. The underlying reason for the mesangial matrix expansion in this biopsy was recurrent IgA glomerulonephritis revealed by IHC and EM. PAS, original magnification x400.

Figure 16. Banff Lesion Score *ah* (arteriolar hyalinosis). A) Banff Lesion Score *ah1* - Mild focal arteriolar hyalinosis (arrow). PAS, original magnification x630. B) *ah2* - Moderate arteriolar hyalinosis (arrow). PAS, original magnification x630. C) Banff Lesion Score *ah2* - Note in this image there is both linear (short arrow) and nodular hyalinosis (long arrow). For a score of *ah2*, more than 1 arteriole displaying moderate to severe is required. Jones silver stain, original magnification x630. D) Banff Lesion Score *ah3* - Severe circumferential arteriolar hyalinosis with luminal occlusion. For Banff Lesion Score *ah3*, hyalinosis of this severity (arrow) must be present in many arterioles as depicted here. PAS, original magnification x630.

Figure 17. Banff Lesions Score *i-IFTA* (Inflammation in areas of interstitial fibrosis and tubular atrophy). Image A shows Inflammation in areas of interstitial fibrosis and tubular atrophy (arrow). This lesion score ranges from 0 to 3, based on the percentage of scarred areas of the cortex (ie, areas qualifying for *ci* and *ct*) involved by inflammation. It is one of the criteria necessary for a diagnosis of Chronic Active TCMR Grade IA or IB. Masson’s trichrome, original magnification x200. B) in contrast shows interstitial fibrosis without significant infiltrate (arrow). H&E, original magnification x400.

Figure 18. Acute Thrombotic Microangiopathy (TMA). A) An acute TMA affecting a glomerulus with fibrin thrombi (long arrows) and fragmented red blood cells (short arrow) in capillary loops. Trichrome, Original magnification x400. B) An acute TMA affecting a small arteriole (arrow). Acute TMA is one of the histological features used as histological evidence of acute tissue injury in Active ABMR. However, TMA is not specific for ABMR and can be seen in eg, recurrent disease or Calcineurin Inhibitor Toxicity. Trichrome, original magnification x400.

Figure 19. Severe Peritubular Capillary Basement Membrane Multilayering (PTCML) as demonstrated by EM. A) This Additional Diagnostic Parameter is a criterion for ABMR chronicity. It is defined as 7 or more layers of basement membrane in at least a single cortical peritubular capillary and 5 or more in at least 2 additional capillaries. This particular capillary shows 8 layers (arrow). Transmission EM, original magnification x14,000. B) This image demonstrates a peritubular capillary with 5 layers of basement membrane (arrow). Transmission EM, original magnification x10,000.

Figure 20. Banff Classification Diagnostic Category 6 (other). These images illustrate some of the more common examples of key lesions specified under category 6. A) Pyelonephritis with neutrophilic casts (arrow) and neutrophilic infiltrates with tubulitis. H&E, original magnification x200. B) BK Virus Nephropathy with typical ground glass intranuclear inclusions as seen on hematoxylin and eosin stain (arrows). H&E, original magnification x400. C) Acute Tubular Injury with widespread isometric vacuolisation of tubular epithelial cells (arrow) associated with acute Calcineurin Inhibitor Toxicity and other forms of injury. H&E, original magnification x200. D) Recurrent glomerulonephritis (membranoproliferative immune complex glomerulonephritis type I in this case) with split glomerular basement membranes (arrow). The diagnosis was confirmed and transplant glomerulopathy excluded by positive IF for immunoglobulin heavy-, light-chains and complement slit products as well as abundant subendothelial electron dense immune complex deposits on EM. PAS, original magnification x400.
